# A Qualitative Study on Civil-Military Cooperation in a Dutch Hospital During COVID-19

**DOI:** 10.1093/milmed/usaf509

**Published:** 2026-04-17

**Authors:** Jacobine Janse, Jori P Kalkman, Adriaan P C C Hopperus Buma, Aura Timen

**Affiliations:** Athena Institute, VU University Amsterdam, De Boelelaan 1085, Amsterdam, 1081 HV, The Netherlands; Faculty of Military Sciences, Netherlands Defense Academy, Postbus 90002, Breda, 4800 PA, The Netherlands; Faculty of Military Sciences, Netherlands Defense Academy, Postbus 90002, Breda, 4800 PA, The Netherlands; Department of Public Health, Netherlands School of Public and Occupational Health, Churchillaan 11, Utrecht, 3527 GV, The Netherlands; Athena Institute, VU University Amsterdam, De Boelelaan 1085, Amsterdam, 1081 HV, The Netherlands; Department of Primary and Community Care, Radboud University Medical Centre, Postbus 9101, Nijmegen, 6500 HB, The Netherlands

## Abstract

**Introduction:**

During the COVID-19 outbreak, military personnel were deployed on an unprecedented scale to overwhelmed healthcare institutions. In the Netherlands too, military medical personnel were deployed to cope with the major patient influx. This study focuses on the civil-military cooperation at the Dutch University Medical Centre Utrecht (UMCU). We explore collaboration experiences and identify lessons for future collaboration.

**Materials and Methods:**

A qualitative study was conducted based on a theoretical framework consisting of network theory, literature on emergent networks, and inter-organizational theory. Data were collected from semi-structured interviews with 34 participants; both civil and military personnel. An abductive thematic analysis was guided by five themes recovered from earlier research on civil-military cooperation. An ethical review and approval for non-medical research was obtained from the Research Ethics Review Committee Faculty of Science of the VU University Amsterdam (BETCHIE 2022.038).

**Results:**

Military assistance at UMCU was perceived to be essential in the COVID-19 crisis and was characterized by its prolonged duration. Day-to-day obstacles arose and were overcome, including varying levels of medical skills and organizational culture differences. Yet, the prolonged duration of the deployment had a severe impact on the collaboration. The common goal became indistinct over time and the collaboration suffered from an ambiguous crisis definition and lacked a clear exit strategy. This uncertainty caused declining motivation amongst personnel.

**Conclusions:**

Military medical support was highly appreciated, but crisis support lacked clearly defined phases to demarcate an end to the crisis, which also hampered the collaboration. Therefore, more attention to the different phases of the Emergency Management Cycle (EMC) is needed. Collaborative actions in the preparedness phase can familiarize partners in an early stage. Distinction between the response and recovery phase can provide transparency on the exit strategy.

## INTRODUCTION

### Military Support During COVID-19

Many countries task their armed forces to support civil authorities during times of crises. For example, during the COVID-19 outbreak, military support was provided to overwhelmed civil healthcare institutions on an unprecedented scale.^1^^,^^2^ In multiple countries, this included military medical support, ensuring continuous care for patients.^3^

**Figure 1.**
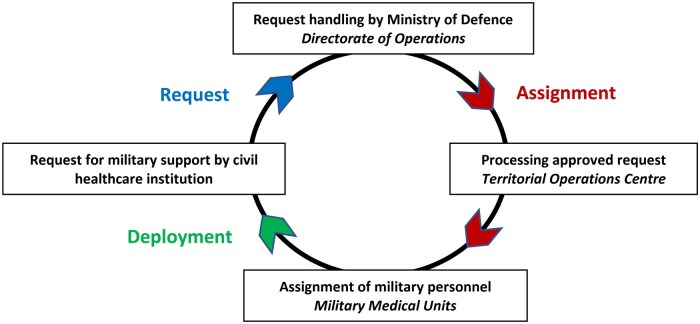
Request-Assign-Deploy loop (RAD-loop). The process of requesting military support by a civilian healthcare institution, shown as a Request-Assign-Deploy loop in study of civil-military collaboration.

In the Netherlands too, civilian institutions can request military support. This process follows a Request-Assign-Deploy-loop (RAD-loop). First, military support is requested by a civil authority, personnel are subsequently assigned, after which they deploy to provide support (see **Figure 1**).

### Research Gap on Civil-Military Cooperation

An extensive literature review by Janse et al on civil-military cooperation (CMC) in outbreak management concludes that papers often list military activities, but lack in-depth empirical observations on collaboration dynamics.^4^ Moreover, most CMC in crises is short-term, so prolonged CMC has remained under-studied. This is problematic since institutions like the World Health Organization (WHO) and North Atlantic Treaty Organization (NATO) are emphasizing the crucial role of long-term CMC in response to future health crises.^5^^,^^6^ The recent COVID-19 outbreak provides an ideal opportunity to assess a long-term civil-military outbreak response. It allows for an in-depth qualitative evaluation on collaboration experiences of stakeholders, providing lessons for future activities.

### Objective and Research Question

This qualitative study aims to explore CMC experiences during the COVID-19 outbreak in the Netherlands and to identify lessons for the future. The study focuses on military deployment of medical personnel to the University Medical Centre at Utrecht (UMCU), the Netherlands. The research question is formulated as follows: ‘How did the main actors in the civil-military network at UMCU perceive their collaboration during the COVID-19 outbreak?’

### Theoretical Framework

This study examines collaboration of stakeholders during an outbreak response, in which different organizations cooperate to reach certain objectives in a hitherto unfamiliar situation. Specifically, this study is based on network theory,^7^^,^^8^ literature on emergent networks,^9–11^ and research on inter-organizational collaboration.^8^^,^^12^^,^^13^ Furthermore, it builds on a scoping review on CMC in outbreak management, which identifies five recurring themes in CMC: Managing relations, Framework conditions, Integrating collective activities, Governance, and Cultural differences.^4^ The characteristics from the theoretical perspectives help to understand the nature and origins of the challenges in each theme. For instance, managing relations requires mutual trust and goal consensus. **Table 1** shows an integration of these theories and its characteristics with the identified themes from the scoping review.

**Table 1.** Integration of Theory Into Identified Themes From the Literature

Themes scoping review	Theory characteristics	Theoretical perspective
Managing relations	Trust, goal consensus	Network
	*Provan and Kenis* ^7^	
	Heterogeneity of links	(Inter)organizational
	*Thévenot* ^13^	
Framework conditions	Division of labor	(Inter)organizational/network
	*Puranam et al* ^12^ *; Kenis and Raab* ^8^	
Integrating collective activities	Network-level competencies	Network
	*Provan and Kenis* ^7^	
	New tasks	Emergent networks
	*Quarantelli* ^9^ *; Kim et al* ^10^	
	Integration of effort	(Inter)organizational/network
	*Puranam et al* ^12^ *; Kenis and Raab* ^8^	
Governance	New structures	Emergent networks
	*Quarantelli* ^9^ *; Kim et al* ^10^	
	Interdependency, (coping with) changes	Emergent networks
	*Drabek and McEntire* ^11^	
Cultural differences	New structures	Emergent networks
	*Quarantelli* ^9^ *; Kim et al* ^10^	

## METHODS

### Study Design and Setting

This study uses a qualitative research approach to gain insight into CMC at UMCU during COVID-19. The Ministry of Defense supported UMCU on 3 occasions between April 2020 and January 2022. During the first wave (April-July 2020), it deployed 92 military personnel to UMCU and also staff to other healthcare facilities. For the second deployment (October 2020-June 2021), the ministry decided to concentrate all military support at UMCU, deploying 150 troops to the hospital. It also required the hospital to serve as a national patient overflow capacity. Lastly, there was a short third deployment of 60 personnel from December 2021 to January 2022. **Table 2** shows the periods and the number of military personnel per deployment. The deployed military personnel consisted mainly of nurses, but also included physicians and support staff.

**Table 2.** Number of Military Personnel Deployed Per Deployment Period

Period of military deployment to UMCU	Number of military personnel deployed
April 2020—July 2020	92
(3 months)	
October 2020—June 2021	150
(7 months)	
December 2021—January 2022	60
(1 month)	

Abbreviation: UMCU: University Medical Centre Utrecht.

Furthermore, the autonomous Central Military Hospital (CMH) is located at the UMCU building and UMCU houses a Major Incident Hospital (MIH). This dormant hospital, a cooperation between UMCU, the Ministry of Defense and the Ministry of Health, can be activated within 30 minutes contingent upon large-scale, multiple casualty incidents.^14^

### Selection and Study Population

A total of 34 UMCU- and military personnel were interviewed. They were either involved in requesting military support and assigning personnel or were deployed to UMCU during the response. We aimed to interview representatives in each of the 3 phases (i.e., Request, Assign, and Deploy) from both the civilian and military side. Since most stakeholders in the collaboration were active in the Deploy-phase, most respondents were active in this phase (**Table 3**). Thus, our sampling was largely purposive. We started data collection by approaching key military actors, requesting referrals to other participants from each of the categories we identified. Saturation was reached when participants started referring to identical key players and no new insights emerged from interviews. In fact, interviewees began to repeat information that we already acquired, at which point we decided to conclude data collection. The number of military staff is relatively high, owing to high military staff turnover on the workplace. The SRQR guidelines were the performing standard for reporting this study (**Supplementary File S1**). Before data collection, all participants received an informative brochure and signed an informed consent form. An ethical review and approval for non-medical research was obtained from the Research Ethics Review Committee Faculty of Science of the VU University Amsterdam (BETCHIE 2022.038).

**Table 3.** Distribution of Participants Over Different Request-Assign-Deploy*-Loop Levels and Between Civilian and Military Participants

	RAD*-loop level			
Participants (*n* = 34)	Request	Assign	Deploy (staff)	Deploy (nurse/physician)
Civilian (*n* = 13)				
Male	2	-	2	0
Female	3	-	1	5
Military (*n* = 21)				
Male	4	3	3	1
Female	1	1	5	3
Total no.	10	4	11	9

* RAD-loop: The phases of a loop-shaped process of requesting for military support by the civilian healthcare institution. Request: the phase of requesting military support by the civilian (healthcare) institution. Assign: the military response by appointing military personnel that fit the civilian request. Deploy: the phase of deployment of military personnel at the requesting civilian institution. Staff: managing staff of medical personnel during the period of cooperation at the hospital.

Abbreviation: RAD: Request-Assign-Deploy.

### Data Collection

For the data collection, semi-structured interviews were used. An interview guide covered the five core themes from our theoretical framework (**Supplementary File S2**). Following a pilot interview in September 2021, data was collected between May and December 2022. Seventeen interviews were conducted in person and 16 interviews were completed online through MS Teams, all by the lead author (J.J.). All interviews were recorded and transcribed verbatim. Interviews were pseudonymized before analysis. Audio files and transcripts were stored in a secure digital environment in line with the data management plan for this study.

### Data Analysis

The data were analyzed by using abductive thematic analysis.^15^ The coding process started with inductive coding of the data, after which an iterative process of recoding and clustering of codes resulted in several main themes. The ATLAS.ti 23.3.4. program was used to assist in the process of coding. The first 3 interviews were coded and analyzed in close consultation with a second researcher (J.P.K.). After discussion and adjustments to the coding framework, this procedure was repeated at different stages. The remaining interviews were coded independently by the lead author (J.J.), yet still in iterative exchange with J.P.K. In case of disagreement, a third researcher (A.T.) was consulted. After concluding the analysis, the results were shared with the participants for a member check.

### Reflexivity

Field notes were written down after each interview to reflect on factors that might have influenced the interview content. As a medical doctor on active military service, the role of the lead author and interviewer was discussed within the research team. The combination of backgrounds allows for integration of perspectives, but might also influence participants and the researcher. For this reason, the role of the interviewer was explicitly stated at the start of each interview and the rest of the author team discussed study outcomes critically to correct for possible bias.

### Role of the Funding Source

The Dutch Ministry of Defense had no role in the study design, collection, analysis, and interpretation of data, nor in the writing of the report, and in the decision to submit the paper for publication.

## RESULTS

The findings present experiences from military and UMCU personnel during their COVID-19 collaboration. The results are organized in five themes. For each theme, when applicable, we discuss the influence of the prolonged collaboration. The ‘Q’ between brackets [Q] including its accompanying number refers to the related quotation in the quote book (**Supplementary File S3**). Before presenting the findings, it is important to note that the general impression of the CMC of a majority of the respondents was positive. Despite shortcomings, participants appreciated the opportunity to collectively participate in the response to the COVID-19 crisis and found a way to work together [Q1, Q2, and Q3].

### Managing Relations

#### History of relations

On the management level, crisis managers connected quickly because of preexisting connections between UMCU and the armed forces. The Central Military Hospital is located at the UMCU and both parties also cooperate in the MIH [Q4]. These connections were used extensively during the first deployment [Q5]. At the workplace level, however, limited pre-crisis relations existed and most assigned military medical units were unfamiliar with the UMCU [Q6]. Consequently, it took time to familiarize themselves with each other’s way of working. Task allocation and responsibilities became clearer over time [Q7]. The military’s decision to deploy a fully new rotation of staff for the third deployment caused problems. Earlier collaboration was not institutionalized or formalized, so the new team felt insufficiently prepared [Q8].

#### Ambiguity regarding the common goal

Initially, the collaboration goal was clear for both sides: coping with an overwhelmed healthcare system during a crisis situation [Q9]. However, during the long-running second deployment, questions arose on when this goal was achieved. Although the common goal suggested that available military support was directly related to the urgency of the crisis, the collaborating parties had to decide when the crisis was no longer a calamity but ‘a new normal’ [Q10]. The unpredictability of the disease complicated this process [Q11]. Moreover, some of the UMCU personnel expected military personnel to stay until the overwhelmed system was recovered, although military managers believed the task to be completed when an agreed patient threshold was reached [Q12, Q13]. In addition, the military lacked a clear exit strategy and had no predefined idea on when and how to end its deployment [Q14]. This was partly caused by the unpredictability of the situation, but was also considered an omission in planning [Q15]. The problems regarding the crisis definition and exit strategy led to indecisiveness, as the collaboration lasted [Q16]. Many participants stated that, despite initial consensus on the common goal, the second deployment lasted far too long for these reasons.

#### Trust and transparency

In general, there was mutual trust among colleagues at both staff and workplace levels [Q17, Q18]. Yet, trust had to grow over time [Q19]. However, trust was undermined by personnel turnover. This was most noticeable for military personnel turnover at the Intensive Care unit, which was perceived as high, causing frustration on the civilian side [Q20]. Despite a general feeling of trust, military personnel at the workplace raised questions on the lack of transparency about the agreements and conditions for military support. For example, it was agreed that both partners would provide 50 percent of personnel, but military personnel felt that they worked more shifts [Q21]. Although UMCU staff recognized this sentiment on occasion, they emphasized that the collaboration was generally characterized by fairness and transparency if the whole picture was considered [Q22].

### Framework Conditions

#### Plans and agreements

Although generic emergency plans on military support to civilian authorities existed in the Netherlands, they were not focused on outbreaks or pandemics [Q23]. University Medical Centre Utrecht, likewise, had no existing plans for scaling up its regular organization. In fact, the authorities had not anticipated a crisis of this magnitude, so there were no preexisting plans to be used. Consequently, the beginning of the collaboration was chaotic and improvised [Q24], but over time the situation improved. This was mainly because of plans being established and adjusted, including training plans for new personnel [Q25]. Financial and legal agreements were made from the onset and did not pose significant barriers in the collaboration. Meanwhile, UMCU has established new plans incorporating lessons from the collaboration [Q26]. The military, on the other hand, has hardly initiated a centralized evaluation to assess and adjust its plans [Q27].

#### Working conditions

On the military side, it was widely believed they worked a disproportionate share of night and weekend shifts [Q28]. Whether or not this is true, it did put pressure on the operational collaboration [Q29]. Although considered a minor distinctive detail, fringe benefits were quoted by a majority of participants. Specifically, the ‘food and beverages’-policy of the armed forces during the first deployment were mentioned. This policy provided military personnel with free drinks and meals while UMCU personnel paid for their own. This situation caused friction as well [Q30].

### Integrating Collective Activities

#### Streamlining clinical skills at the workplace

The teams at the workplace consisted of a pluriform group of medical professionals. Military personnel were newly deployed at the hospital and UMCU personnel often came from various departments or flex-pools. Individual skills levels of both military and UMCU personnel diverged significantly. Expectations among UMCU personnel with regard to military nursing skills, were not always met [Q31]. Particularly, a lack in clinical nursing skills was observed [Q32]. To resolve this, effort was put in to designing training programs and the introduction of a skills classification system by color, visible on each person’s name tag [Q33][Q34]. It was perceived that, given the circumstances, this enabled the highest possible quality of care provision [Q35].

#### Motivation

Civilian and military participants differed in their personal motivations. University Medical Centre Utrecht personnel indicated working from a perspective of intrinsic motivation on high quality patient care in a hospital setting, although the military personnel worked from a more instrumental perspective, practically fulfilling a military task [Q36]. Some military personnel even argued that they preferred not to work in a regular hospital at all [Q37]. At the start of the collaboration though, most personnel were strongly motivated to cope with the crisis [Q38]. Motivation of military personnel declined over time, mainly as a result of the lack of transparency on collaboration agreements and indecisiveness on the exit strategy [Q39, Q40]. The length of the deployment also caused a strong feeling among military personnel that it came at the expense of regular duties and their organization’s (combat) readiness [Q41].

### Governance

#### Working apart together

Although UMCU had final responsibility for the collective efforts, a clear separation of command between partners existed throughout all 3 deployments. The military brought in their own staff to maintain command of deployed personnel [Q42]. Although they communicated closely with UMCU counterparts, they remained separate from the UMCU organization, hampering communication [Q43] and causing incomprehension [Q44]. Overall, the governance process was time-consuming as a result.

### Organizational Differences

#### Challenges in cultural differences

Organizational differences caused obstacles in the collaboration. For instance, UMCU personnel were unfamiliar with military hierarchy, resulting in difficulties in finding their appropriate counterpart [Q45]. Military personnel have a self-perception of being bold, straight and decisive, but were sometimes perceived as rigid and overbearing by UMCU personnel. University Medical Centre Utrecht personnel, on the other hand, think of themselves as academic, care-oriented and inquisitive, but military partners occasionally perceived a sense of superiority, a pampering attitude towards patients, and slow decision-making processes. This caused mutual incomprehension. Most prominently, military personnel were sometimes criticized for being too blunt [Q46], although UMCU personnel were at times seen as indecisive and inefficient [Q47].

#### Opportunities in cultural differences

Differences also created opportunities [Q48]. University Medical Centre Utrecht participants called military personnel a valuable addition and were inspired by their energy [Q49]. Also, the military concept of *runners* was adopted by UMCU [Q50]. Runners are non-medical staff supporting medical personnel. Military staff, in turn, appreciated gaining knowledge on the financial side of healthcare and hospital management [Q51].

## DISCUSSION

This study was designed to explore collaboration experiences and to identify lessons learned in civil-military collaboration in a Dutch hospital during the COVID-19 outbreak. The prolonged military support was unique compared to previous domestic deployments. In general, most participants were positive about the collaboration. Although a number of day-to-day practical issues had been overcome, the lack of clear criteria for the exit strategy and an unclear crisis definition led to indecisiveness and perceptions of an unnecessarily long deployment. A perceived lack of transparency and the feeling that the deployment came at the expense of regular military activities caused declining motivation on the military side.

### Impact of Unclear Definition of Crisis

In most countries, military support during crises is a *last resort* option, meaning that all other (civilian) options need to be tried first.^16^ This study showed that when a crisis situation is not clearly defined and when it is difficult to demarcate an end to the crisis, perceptions and expectations concerning collaboration may diverge. For example, the military partner planned to downscale and end the support when patient numbers declined, although the civil partner expected continuing support to recover from an overwhelming situation. For the latter, it was only after the recovery period that the crisis was (truly) over. Despite initial agreement on the need to cope with patient flow, the collaboration lacked long-term goal consensus. This reflects the theory of Provan & Kenis on goal consensus in successful collaboration.^7^ The partial goal consensus caused an ambiguous exit strategy, preventing clear decision making. Therefore, the duration of collaboration was stretched, which caused uncertainty and frustration. In line with the findings of Kenis & Raab the ‘reward’ for cooperation declined, reflected in declining motivation for the collaboration.^8^

To improve exit strategies, the emergency management cycle (EMC) is proposed here as a tool for analyzing crises.^17^ The emergency management cycle defines crisis management as having five phases: prevention, preparedness, readiness, response and recovery. During the COVID-19 response at the UMCU, there appeared to be little awareness of distinctions between these phases, and the distinction between the response and the recovery phase in particular. If the EMC phases are acknowledged and recognized, the roles per phase can be defined, supporting an exit strategy appropriate to the requirements of that phase. In this way, the exit strategy can be predetermined and justified.

### Impact of a Different Medical and Organizational Background

The day-to-day practical issues of the collaboration originated mainly in personnel having different levels of medical skills and the organizations being culturally different. The clinical competencies of military nurses and physicians did not always meet the expectations of their civilian colleagues. This study does not aim to evaluate the skills levels of personnel, but it is worth exploring possible explanations and solutions nevertheless. In the Netherlands, military medical personnel are primarily trained for trauma care, but practical exposure is limited. Furthermore, the characteristics of a COVID-19 patient are not those of a wounded soldier on the battlefield. A certain mismatch of skills during COVID-19 was therefore to be expected. Nevertheless, civil support is a core responsibility of the armed forces, also in health crises. This should be taken into account during the preparedness phase. It requires a clear strategic and political choice to ensure that military medical personnel are ready to support civilian healthcare providers in a range of scenarios. Next, the collaborating partners were required to get acquainted and overcome cultural differences in the midst of a crisis. University Medical Centre Utrecht personnel was confronted with the inward-focused military culture,^18^ and military personnel had to navigate an unfamiliar academic environment. Pre-crisis relations at all levels and the exchange of organizational knowledge could have saved precious time and could have avoided incomprehension during the crisis. This requires pre-crisis commitment to investing in the relationship.

### Potential Impact beyond This Study

The COVID-19 pandemic showed the world’s vulnerability to health crises. Global developments, such as emerging diseases as a result of climate change and the expansion of antibiotic multidrug resistance, pose potential challenges to healthcare systems worldwide.^19^ Although hitherto unprecedented, the scale of military deployment during COVID-19 indicate that armed forces may play a significant role in future health crises too.^20^^,^^21^ Therefore, this study is valuable in preparation of future civil-military collaboration in health crises.^22^ Furthermore, the results of this study might even be applicable to other areas of long-term collaboration in healthcare, for example the WHO One Health concept.^23^ A scoping review on enabling factors of effective One Health collaboration demonstrates that limited literature is available that describes successes, challenges and lessons learned in operational One Health.^24^ Experience from an adjacent professional field, such as reported in this study, can offer further in-depth understanding of collaborative processes in healthcare.

### Further Research

This paper reports on the perceived experiences of prolonged CMC. Yet, it is to be expected that the CMC also has had its impact on both organizations. With the increasing likelihood of future long-lasting health crises, it is crucial to gain further knowledge on the sustainability of prolonged collaboration between crisis response partners. Currently, many NATO countries, including the Netherlands, are preparing for a potential large-scale conflict. As attention is redirected to medical CMC in case of high numbers of battlefield casualties, many lessons on CMC during pandemic outbreaks may be forgotten. Further research is needed to study this concern.

### Strengths and Limitations

This research is conducted on a specific area of collaboration and provides in-depth insight into CMC mechanisms of in-hospital collaboration during crises. Moreover, it includes collaboration on different organizational levels, representing different views and experiences within the same organizations. Furthermore, this study is conducted within 12 months after the last deployment. It provides a retrospective view in less turbulent times after participants had time for due reflection, but it is therefore also vulnerable for recall bias. Moreover, it is limited to one country and one hospital only, so further research might use a broader sample. Future research might also zoom in on one of the three phases to delve deeper into collaboration enablers and obstacles.

## CONCLUSIONS

This study provides an analysis of the experiences of personnel engaged in CMC in a Dutch hospital during the COVID-19 pandemic in an attempt to improve our knowledge on prolonged CMC. The study demonstrated that pre-existing relationships can facilitate effective collaboration. Moreover, indecisiveness regarding the termination of the deployment and exit strategies led to uncertainty and declining motivation. Using the EMC can provide a clear structure in CMC by marking phases, leading to shared goals and commitments per phase. Additionally, collaborative actions in the preparedness phase can familiarize partners with each other in an early stage. If armed forces are considered a crucial partner in future health crises, CMC requires strategic and political commitment to investments to optimize preparedness and achieve the best response in times of crises.

## Supplementary Material

usaf509_Supplementary_Data

## Data Availability

The data underlying this article cannot be shared publicly because of the privacy of individuals that participated in the study. The data will be shared on reasonable request to the corresponding author.

## INSTITUTIONAL CLEARANCE

Institutional clearance does not apply.
