# Supplementary material for: A Qualitative Study on Civil-Military Cooperation in a Dutch Hospital During COVID-19
Source: Mil Med. 2026 Apr 17;191(5-6):e1241–7. doi: 10.1093/milmed/usaf509 (PMC13143289; doi:10.1093/milmed/usaf509)
Supplement: usaf509_Supplementary_Data [file usaf509_supplementary_data.zip › S2_Interview guide and context.docx]

**Supplementary file 2: Interview guide and context**

**A qualitative study on civil-military cooperation in a Dutch hospital during COVID-19**

This current document describes the interview format and its context for a qualitative study based on interviews exploring experiences from the civil-military cooperation at the UMCU during COVID-19.

**Introduction**

During the COVID-19 pandemic military personnel supported overwhelmed civil healthcare institutions in order to continue civilian healthcare. To receive military support civilian institutions have to request for support. In this process loops emerge that follow the route of: the requesting civil institutions, the receiving military organization, processing of the request, tasking military personnel and finally deployment of military personnel at the requesting civil institution. Proposed as the Request-Assign-Deploy loop (RAD-loop). One of the requesting institutions during the COVID-19 pandemic was the UMCU.


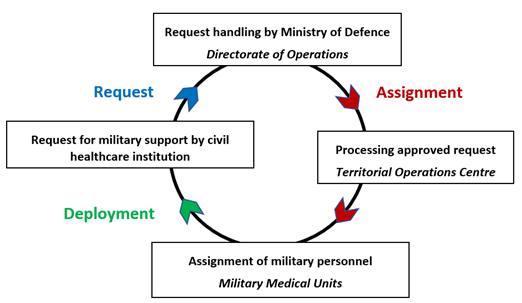


Fig 1. Request-Assign-Deploy loop (RAD-loop)

**Conceptual framework**

The PhD thesis is initiated by a scoping review on existing peer-reviewed literature*.^1^* The scoping review shows five recurring themes in literature related to civil-military cooperation in the management of infectious disease outbreaks.

- Managing relations
- Framework conditions
- Integrating collective activities
- Governance
- Civil-military differences

Several theories relate to the effectiveness of cooperation, and provide also the theoretical backbone of the interviews. Based on the provided scoping review outcomes and theoretical framework, this research focusses on trust and tensions, relations, expectations, integration and civil-military differences related to the civil-military cooperation during the COVID-19 response at the UMCU.

- Formal procedures (theory and practice)
- Informal procedures (practice)

Theories on effectiveness of cooperation

- Network theory: **Network as a variable**

Provan and Kenis (2008)^2^

**Organizational network response**

Kenis and Raab (2020)^3^

- Emergent networks: **New structures with new tasks**

Quarantelli (1983)^4^; Kim (2022)^5^

**Urgency, interdependency, changes**

Drabek and McEntire (2003)^6^

- (Inter)organizational: **Heterogeneity of links**

Thévenot (2001)^7^

**Division of labour & integration of effort**

Puranam et all (2018)^8^

**Research question**

‘Who are the actors within the civil-military network during cooperation at the UMCU and how do they experience their cooperation at the UMCU during the COVID-19 outbreak?’

**Sub questions**

How started the cooperation?

How developed the cooperation in time

What are the experiences (qualitative) regarding the themes from the identified literature?

**Type of research**

Explorative, qualitative research

**Target group**

Civilian and military stakeholders engaged to (one of) the phases of the RAD-loop for civil-military cooperation during the COVID-19 response. The civil institution is the UMCU.

**Methodology**

Qualitative research by semi-structured interviews. This way a balance can be found between cohesion and reproductivity while at the same time there is room to explore new dynamics not yet found (in literature).

**Instruments**

(Explorative) semi-structured interviews, field notes (possibly focus group). Full transcription of all interviews (and focus group meetings) will be performed. Transcripts will be analysed by thematical coding in order to identify themes from the data (by Atlas.ti or MAXQDA).

**Topic list ordered by theory**

| **Theory** | **Question(s)** |
| --- | --- |
| Formal and informal procedures/contacts | - How has civil-military cooperation establish during COVID-19?  - When did (in)formal contact/procedure play a role? |
| **Network theory**  Network as a variable  Provan and Kenis (2008)^2^  Organizational network response  Kenis and Raab (2020)^3^ | - Which parties were involved in this civil-military cooperation? For which tasks?  - What was the purpose of Defence/UMCU to collaborate?  - Did the civil/military organization have the same ideas about this? Common goal/consensus?  - Clarity about tasks, roles and mandates?  - Deployed at the right competency level?  - Was there mutual trust? What does that show?  (trust, number of network participants, network goal consensus and need for network-level competencies) |
| **Emergent networks**  New structures with new tasks  Quarantelli (1983)^4^; Kim (2022)^5^  Urgency, interdependency, changes  Drabek and McEntire (2003)^6^ | - How has civil-military cooperation establish during COVID-19?  - How were organizations (civ/mil) prepared for collaboration? Plan?  - What did the framework conditions look like (present? clear? E.g. money, legal framework?)  - Clarity about tasks, roles and mandates?  - How was the contact?  - How did the information provision proceed?  - How has the collaboration developed over time?  - Ability to learn and adapt within the crisis |
| **(Inter)organizational**  Heterogeneity of links  Thévenot (2001)^7^  Division of labor & integration of effort  Puranam et all (2018)^8^ | - Was there cooperation/knowledge of each other (in other areas) before the COVID-19 outbreak?  - Clarity about tasks, roles and mandates? Invested in the right place?  - What did you think about the collaboration (motivation)?  - How did the information provision proceed?  - How was knowledge and/or data shared within a partnership (trust?)  - How did the mutual contact go? |
| **Outcomes Scoping Review^1^**  Managing relations  Framework conditions  Integrating collective activities  Governance  Civil-military differences | - Was there cooperation/knowledge of each other (in other areas) before the COVID-19 outbreak?  - What were the expectations of collaboration? What came of that?  - What did the framework conditions look like (present? clear? E.g. money, legal framework, agreements?)  - How was the contact?  - How did the information provision proceed?  - Clarity about tasks, roles and mandates?  - Were there (cultural) differences? How bridged?  (successful cooperation requires sustainable relations, binding agreements, transparency, a clear operational perspective and acknowledgement of organizational cultural differences) |

**References**

1 Janse J, Kalkman JP, Burchell GL, et al. Civil–military cooperation in the management of infectious disease outbreaks: a scoping review. *BMJ Global Health* 2022; **7**(6): e009228. doi:10.1136/bmjgh-2022-009228.

2 Provan KG, Kenis P. Modes of network governance: Structure, management, and effectiveness. *Journal of Public Administration Research and Theory* 2008; **18**(2): 229–252. doi:10.1093/jopart/mum015.

3 Kenis P, Raab Jr. Back to the Future: Using Organization Design Theory for Effective Organizational Networks. *Perspectives on Public Management and Governance* 2020; **3**(2): 109–123. doi:10.1093/ppmgov/gvaa005.

4 Quarantelli EL. Emergent behavior at the emergency time periods of disasters. 1984. Ohio State Univ Columbus Disaster Research Center.

5 Kim Y, Lee K, Oh SS, Park H. Effectiveness of Emergent Ad Hoc Coordination Groups in Public Health Emergencies. *Risk Anal* 2022; **42**(1): 5–20. doi:10.1111/risa.13751.

6 Drabek TE, McEntire DA. Emergent phenomena and the sociology of disaster: Lessons, trends and opportunities from the research literature. *Disaster Prevention and Management: An International Journal* 2003; **12**(2):97–112. doi:10.1108/09653560310474214.

7 Thévenot L. Organized Complexity: Conventions of Coordination and the Composition of Economic Arrangements. *European Journal of Social Theory* 2001; **4**(4):405–425. doi:10.1177/13684310122225235.

8 Puranam P. The microstructure of organizations. Oxford University Press 2018. ISBN: 9780199672363 (ISBN).
