# Supplementary material for: A Qualitative Study on Civil-Military Cooperation in a Dutch Hospital During COVID-19
Source: Mil Med. 2026 Apr 17;191(5-6):e1241–7. doi: 10.1093/milmed/usaf509 (PMC13143289; doi:10.1093/milmed/usaf509)
Supplement: usaf509_Supplementary_Data [file usaf509_supplementary_data.zip › S3_Quotebook-revision.docx]

**Supplementary file 3: Quotebook**

| *Q1* | *Deployments are always tricky and never without glitches but in general people truly appreciated being able to contribute. [R8-mil-female]* |
| --- | --- |
| *O2* | *In a sense, we were condemned to each other. That could have turned out negatively but sharing this unreal and exceptional experience created a team, I think. [R19-civ-female]* |
| *Q3* | *I think we discovered that we can cooperate very well, that differences are not that big after all. […] that we all want the same even though we behave slightly different. I think that is something to cherish. [R22-civ-female]* |
| *Q4* | *The CMH has an intensive collaboration with the UMCU indeed. That has always existed and the lines of communication are short. So, when they started setting up their crisis coordination team and their crisis policy team, they immediately thought: 'and then we also want the CMH there'. [R3-mil-female]* |
| *Q5* | *During the first COVID period, we organized everything in accordance with the Emergency Hospital Principles. […] And the first COVID period was under our [MIH] responsibility, whereby we received personnel from the UMC and from the Defense Organization. [..] In COVID two and COVID three, the Emergency Hospital was used, there was military deployment, but [...] we were just landlords. [R10-civ-male]* |
| *Q6* | *What was very new for us indeed is that we were not doing that with the staff of the CMH. […] But in this case, it were the boys and girls of an operational medical unit. They are not used to the UMCU at all.[R2-mil-male]* |
| *Q7* | *The learning ability […] the second time was a lot easier. Because on our side, we knew how to direct the organization. We knew who should be responsible for what. And we knew which tasks we could delegate to Defense and which tasks lay with us. So, both organizations learned quickly. [R26-civ-male]* |
| *Q8* | *That [recording knowledge from an earlier period] was a really tricky topic. […] From the start it became painfully clear that the only one who had done this before was a corporal who did the administrative side, so to speak. […] I was also surprised that none of my predecessors took the trouble to draw up a script. [R16-mil-male]* |
| *Q9* | *With the hospitals in the first wave, it was really clear to everyone: The military is coming here to ultimately ensure that the chaos and crisis in this hospital can be controlled. [R11-mil-male]* |
| *Q10* | *Such a crisis will of course be phased out at some point. […] At a certain point we have fewer patients there, so a crisis develops into normal care. There is no hard cut. At some point there is a start. Then you arrive in the middle of- And the end is of course much more gradual. So, when is that? [R29-civ-female]* |
| *Q11* | *However, things started to fluctuate a bit then. And you thought: yeah, the crisis is getting better, yes. And then we just hung around and hung around. 'You never know, and who knows, and maybe something will come and'. But yes, at some point that stops, in my opinion. [R6-mil-female]* |
| *Q12* | *We [UMCU] ultimately aim for good care and safety and the military is about: but this is not my job. […] And this no longer seems nationwide, so we [Military] are now going to withdraw. But if you withdraw now, we will have a very major security issue, so we have to do that gradually. But now it's done, like that. So that- […]. The procedure says, A, B, we're here now, so now it stops. I’m like, the procedure may say that, but tomorrow the situation may be different, so you have to keep looking at the whole picture. But the procedure says until here. [R20-civ-female]* |
| *Q13* | *If you say on Monday: we won't be there from Thursday. Yeah, well, for us that is: yes, well, our assignment is over and we are going to do fun things again in our own activities and we can prepare ourselves, we can work with that. We would be euphoric to do it this way again, because it is not without reason that we choose the military. And they don't understand that, they don't want to understand that either and they think: but if you just stay now, we can all take a holiday together and then we can all restart the care, then we can the regular-. So, I can imagine that the UMCU staff is also like: hmm, yes, there is kind of ’Exercise Dust Cloud’, they are gone, and here we are. Even though it's not finished yet. [R25-mil-male]* |
| *Q14* | *I found the criteria on the basis of which you do this [phasing out] to be difficult. What do you use in such a case? Is it the bed occupancy? Is it the number of sick? Is it how many beds are occupied throughout the Netherlands? How do you take into account the future? We have had very erratic predictions of the future. [R27-mil-female]* |
| *Q15* | *[...] that is often the strength, I think, of phasing within a military order. Because you only go to the next phase when the first phase, or at least the previous phase, has been completed. We always do that, except in civil-military cooperation. […] When we start doing that, we don't do things that we normally do. That's weird. [R25-mil-male]* |
| *Q16* | *And that in the end - I also think that rather, suppose this happens again and it is nearing the end, that you think, are we going to get out or not? That decisions also need to be made sooner. Now it took a very long time before the decision was made that we are going to stop working with the military because we can now handle it ourselves. [R30-civ-female]* |
| *Q17* | *When you have a crisis, you still trust each other to do the best for that person. And I think everyone had that attitude. So that is, I think - And how you get there, you have to figure it out with each other on the spot. [R31-civ-female]* |
| *Q18* | *At my table, I think that [trust] developed very rapidly. Because, well, you work together and you know very quickly what you can do with each other and I think that, I also just felt like one of the team. So, at the CCT table I think it was fine. [R3-mil-female]* |
| *Q19* | *[During] the second wave there was a real team in the end, which we all experienced as very positive. The soldiers and us and there was certainly trust, yes, certainly a lot. And of course, there are always people in the team, [...] but overall, I can say that in the end we certainly trusted each other. R19-civ-female]* |
| *Q20* | *What I found very difficult about that is that you had, for example, three weeks where you thought, I'm going to get to know you, and then the whole thing was changed again and then you had people who had zero experience. And then I could start explaining everything again. [...] And I thought, how can you change really everything again? That was the military’s planning. Then whole pots of new people were opened, which I found super frustrating. […] All new people where you [previously] knew what you could entrust to them, what you could ask of those people, suddenly gone again. So, it started all over again for me and that took a lot of energy. [R31-civ-female]* |
| *Q21* | *And so you actually, off the top of my head I think it was 50% of the capacity in a nursing department the we provided. But that means 50% and no more. That has also been done regularly. [...] But there too, structurally, we were in a kind of overcapacity. So, we pretty much ran the department. [...] They [UMCU] simply pulled people away from the UMCU, that was the main reason. It may happen once because you have an acute illness, then it is not a problem, but it became really structural. And we really reacted against that and really indicated: yeah, that is not possible. [R6-mil-female]* |
| *Q22* | *And we have always been very transparent about that, [...] but that felt different, because I also got that back, it felt different. […] So, if indeed there are five day shifts and you only need three, then in the beginning some of the UMC did indeed return to the department, because the departments had a crisis in the first place. And in addition, we also said that the Ministry of Defense would be out and UMC would remain and so on... But that transparency about it at nursing level, department level, I don't think you work at the bedside, is transparent enough. Because they see different things, because they only see one department, one unit and they don't see three or four units, they don't know. [R20-civ-female]* |
| *Q23* | *There is [a plan] in the field of-. Look, the system of safety regions and that national system, when you talk about civil-military, that is there. That's just there. Just not very specifically to this, a pandemic. There just isn’t. Which is very strange, because if you look at the two biggest risks that we have defined in the Netherlands, they are about flooding and about a pandemic. We have prepared for a flood, but not for a pandemic. [R1-mil-male]* |
| *Q24* | *In that first wave, everything had to be devised on the spot. I mean, we didn't know, you don't know at all which way to go and how we should have meetings, how we should interact with each other. You do not know. [R20-civ-female]* |
| *Q25* | *So we created a training program for employees who were going to be deployed to the ICU, which at first was two hours, mind you, two hours, and then a few months later it was a two-day program. [R26-civ-male]* |
| *Q26* | *So if a crisis were to break out now, we also have a battle plan, a cooperation plan with Defense. […] So that's there now. But we had to figure that out. [R20-civ-female]* |
| *Q27* | *[This assessment] is the very first time that [UMCU deployment] is put on paper of how that happened. Funny enough, there is apparently no interest in this within the [military] organization, at least I didn't notice it. And if it is marked, then it is at TOC level, DOPS level, whatever. But people simply, de facto, completely forget the levels where the collaboration with civil society actually happened. [R33-mil-male]* |
| *Q28* | *We worked all holidays, weekends, we worked all the time. And they also had holidays every now and then. And we didn't have a holiday either, […]. At a certain point that causes friction. Or at least, not so much among ourselves, because they can't do anything about it either. They don't decide it, but it did cause friction to a higher level. And people really had an opinion about it. And people [at Defense] were also ready for a holiday and things like that, so that did something. [...] And you don't want to call in sick, because you don't want to disappoint a colleague, because then you know that he or she will have to work extra again. But yes, that certainly did something to people yes [R6-mil-female]* |
| *Q29* | *Those soldiers also realized: Hey, when it's night, we always have the night shifts, where are the-. So the feeling that you are not being treated equally comes into play. The feeling quickly arose that if there was a midwatch, the military would be there and the UMC colleagues might be treated a little more friendly. […] That was demotivating. [R3-mil-female]* |
| *Q30* | *I thought the biggest cultural difference was that MOD has to take care of its personnel and the UMC does not, which of course also has to take care of the personnel, but-. In the beginning, Defense saw it as, let me phrase it correctly, as a mission. So then you have to take care of your staff, in terms of catering, accommodation and things like that. And you noticed that it was a cultural difference and that it also clashed at some point. The military received meals, the UMC had to bring its own sandwiches. [R21-civ-male]* |
| *Q31* | *[…] those [military] nurses were basic trained, so we assumed that they all had the same education, including nurses from Defense […] That was not always the case, let me put it this way. [R22-civ-female]* |
| *Q32* | *And of course, you also have that with the Registered Military Nurses, they receive a lot of theory, they are trained quite well in the military, but then to the authorized, to the competence, to the regular civilian care, for example, there is a gap. And that gap is that they know it theoretically, but in practice it is always slightly different. And then you think you know it, but you actually don't have enough experience. [R21-civ-male]* |
| *Q33* | *Well, at first the UMC was also quite negative. Because they were like 'yeah, what can they do actually?'. So, there was already a pre-tension of: 'do we want this' and 'our care is important and [of] a higher level'. And then we put them through a kind of training for 2 days. […] And after a very steep learning curve, I think our colleagues really were liked and everything went well. [R3-mil-female]* |
| *Q34* | *So people had dots and colors, to the extent they could or could not do something. And we also had nurses from the UMC, we also gave them numbers and a one, two, three, four, five. What can you do and the military people also had a sticker on an extra pass, to show what the qualifications, capabilities are and what people can and cannot do. [R20-civ-female]* |
| *Q35* | *There is quite a bit of criticism about the level of knowledge and skills of military medical personnel. Military personnel do not always recognize this in themselves, but everyone has their blind spot, including the anesthesiology staff who come from the operating room and now do things in the ICU. […] Yet something good has been done together. We worked hard, sometimes with insufficient knowledge. Patients have undoubtedly suffered from this, but the outcome is many times better than if these efforts had not been made. It is a crisis, which means working in different circumstances. [R26-civ-male]* |
| *Q36* | *A [civil] nurse here [in the hospital] who says: this is my profession, my work, my dream my x. And a soldier says: I'm just being posted here, I don't think anything of it for the time being, because I think this is stupid care. Fine, but I serve this in the national interest. It sounds a bit high, but that's why I do it this way. […] I have an assignment. But my job is this and I have the assignment that, so there is something intrinsically different behind it. [R20-civ-female]* |
| *Q37* | *Of course I was disappointed [that I was appointed]. Finally finished with the [military] training, you can start doing military things. And then you are put in the hospital. [But] you know, the military has that option. [...] And you don't want to - I wouldn't be so quick to object, because you know, well, the work has to be done. If I don't go, there will be someone else who will have to stay longer. [R34-mil-female]* |
| *Q38* | *I think that in the end we had a team that was very motivated and if you eventually see that something really good arises from such a crisis situation, because that is what happened anyway, then you are all very motivated. So we had a very motivated group. [R19-civ-female]* |
| *Q39* | *In that second wave, of course, at a certain point we started to function more and more like a normal department and therefore I think it was very understandable that at a certain point the soldiers said, but it is now seventy and thirty percent, we are now only thirty percent UMC nurses and at one point that ratio was also somewhat gone. And I think they were right about that, that there had to be- […] so I understand very well that at one point the soldiers were like, hello, where are the UMCU nurses and what are we supposed to do here? And I understood that very well, indeed. [R19-civ-female]* |
| *Q40* | *But in that second wave it was after about six or seven months- [...] Around the holidays things started to weaken a bit and it became- Should they have a new- There was already talk about them getting out, but it was extended again and again by a few weeks. And I noticed that they were less motivated, you just noticed that they had less job satisfaction and that reflects on a department. [R30-civ-female]* |
| *Q41* | *I think it [civ-mil cooperation] can work very well, but it also causes a lot of disruptions for an operational unit in the long term. We are actually intended as 400 [medical battalion] to prepare role 2 [hospital] capacity for deployment. And because we have provided corona support, we have simply not done anything green [military] for I think 1.5 years. So a lot of that knowledge and skills have actually disappeared. […] And they [Defense] have also acquired white [civilian] knowledge, but not always in the right field. [...] In the long term it has cost us a lot. It did yield something, because we were in the picture for a while and people were able to keep up with their white (civilian)skills, but I didn't really think about the whole aftermath. [R13-mil-female]* |
| *Q42* | *We have set up contact points there [at hospital staff level] from our [Defense] organization: detachment commanders who managed. […] and they did their own planning, which allowed them to monitor it a little better. And that's quite smart and it was explained to them. Culturally, this is of course not very well accepted, because people prefer: we decide the shots here, right? Indeed, but you ask for our involvement. [R28-mil-male]* |
| *Q43* | *I thought, this is apparently how it works. I'll just leave it like that and I'll take a tour of Tom, Dick and Harry to see who I should contact. But a bit of simplification would be possible - Then it would fit better the structure we have in the UMC Utrecht and then it would also fit in more easily. Then you just have a chief nursing team and a chief doctor team and maybe one boss above them. And that's it. And now I found it complicated sometimes. [R29-civ-female]* |
| *Q44* | *But what I found complicated is your [Defense] decision-making process to achieve this. So, at the end we now actually know each other when things will get stressful. […] I think during the last wave, […] I thought that a bit of a situation had arisen that by the time you were deployed, you saw that the peak had already practically gone away. And that you actually started here too late, so you were not very effective and neither did your people have the feeling that they were deployed just as effectively. [R18-civ-male]* |
| *Q45* | *It really bothered me every time, so I found it difficult to know who I was talking to. Is it a doctor, is it a nurse, is it someone- And regardless even of ranks, I still find that incredibly difficult in that green uniform. And then there were a lot of changes, so I still don't understand whether the commander or the colonel is in charge. I never played Stratego, I always said, but that didn't help either. […] But […] it took a really long time before I had a number of people, you can, I can discuss this with you. Then they said, you shouldn't discuss that with me, you should discuss it with- Then I first get a position and then I said guys, give me a name, because maybe that will help me get further. [R24-civ-female]* |
| *Q46* | *Military personnel have a slightly different behavior than- [...] There are of course many soldiers who do the Registered military nurse training, they are military personnel and so they remain military and they suddenly become nurses. And the UMC has people who are doing nursing training and have a real caring side. And that is slightly different on the military side. There are- [Military personnel are] harder, easier, less caring, faster. And you have to think about that. […] No, [that] is not negative at all. [21-civ-male]* |
| *Q47* | *[Recognition] Of that academic culture, absolutely. And that especially among internists. But at the end of the day, everyone knows it's about caring for the patients, so you'll find a mode. But as general military doctors we have indeed sometimes cursed internists when they came up with something that was especially unpractical, especially not pragmatic and actually not essential for the patient.[R34-mil-female]* |
| *Q48* | *I think that these different cultures reinforce each other and that you should preserve the beauty of it, green and white together. [R14-civ-female]* |
| *Q49* | *It just really reinforced each other; those differences reinforced each other because they were so different. So that gives a degree of surprise, amazement and equal learning efficiency, so to speak, and fun. You do it this way, well we do it this way. That. Sounds a little weird, but- That's exactly what you think, okay. And that also gives a measure of immediate energy. [R20-civ-female]* |
| *Q50* | *That whole buddyship, that cooperation with each other, working towards a certain goal with each other- […] And now you see again that the added value of support and that this is not per se necessary [only highly trained healthcare staff], that you can provide better care together.*  *[…] We already started with that [use of non-medical staff], but I think the insight has been strengthened that it is very nice and that there are benefits. [R22-civ-female]* |
| *Q51* | *For me personally, I have encountered too many times that I did not take money into account. And the influences of money. And I have become more aware of that. Thus, the competency cost-conscious. And that was never my strong point, if I'm completely honest. And I am now at least aware of what we do, how much does it actually cost? And how can we deal with that differently? [R25-mil-male]* |
